# Sputtering-Power-Ratio Engineering of SS–Ti–N Coatings: Correlation of Composition, Structure, Morphology, and Hardness

**DOI:** 10.3390/ma19214439

**Published:** 2026-10-22

**Authors:** Faisal Alresheedi

**Affiliations:** Department of Physics, College of Science, Qassim University, Buraidah 51452, Saudi Arabia; f.alresheedi@qu.edu.sa

**Keywords:** thin films, stainless steel, crystal structure, mechanical properties, hardness

## Abstract

Reactive magnetron sputtering offers an effective route for tailoring the composition and properties of hard nitride coatings through controlled adjustment of deposition parameters. In this study, stainless-steel–Ti–N (SS–Ti–N) coatings were deposited by reactive magnetron co-sputtering from stainless-steel (SS) and titanium (Ti) targets in an Ar/N_2_ atmosphere. The influence of the SS/Ti sputtering-power ratio was systematically investigated by maintaining the Ti target power at 170 W while reducing the SS target power from 170 to 100 and 70 W. The chemical composition, crystal structure, cross-sectional morphology, surface roughness, and hardness were evaluated using X-ray photoelectron spectroscopy, X-ray diffraction, scanning electron microscopy, stylus profilometry, and micro-Knoop hardness testing, respectively. XPS revealed increases in Ti and N concentrations from 4.25 to 14.0 at.% and from 35.6 to 47.7 at.%, respectively, as the SS target power decreased. XRD revealed an fcc-based, nitrogen-rich structure with changes in lattice spacing, while SEM showed an evolution toward a more uniform and compact columnar morphology. These changes were accompanied by a decrease in surface roughness from approximately 0.012 to 0.005 µm and an increase in hardness from 2450 to 3198 kgf/mm^2^ (approximately 24.0 to 31.4 GPa). Among the investigated coatings, the sample deposited at an SS/Ti target-power combination of 70/170 W exhibited the highest Ti and N concentrations, the lowest surface roughness, and the highest hardness. These findings demonstrate that adjusting the relative sputtering powers provides a practical means of tailoring the composition, structure, morphology, and mechanical properties of SS–Ti–N coatings, highlighting their potential for protective surface-engineering applications.

## 1. Introduction

Austenitic stainless steels, particularly AISI 304 and AISI 316, are widely used in engineering applications because of their excellent mechanical strength, toughness, corrosion resistance, and chemical stability. However, their relatively low surface hardness and limited wear resistance can restrict their performance under severe friction and mechanical contact. Nitrogen-based surface modification has attracted considerable attention as an effective approach to improving surface hardness and wear resistance through nitrogen incorporation and lattice expansion. Several techniques have been developed for this purpose, including ion implantation, plasma immersion ion implantation, plasma nitriding, and thermochemical treatments [1,2,3,4,5,6,7,8]. Reactive magnetron sputtering provides an alternative route for producing nitrogen-containing stainless-steel coatings with controlled composition and microstructure [9,10,11,12,13,14,15]. By incorporating nitrogen during coating growth, this technique enables the development of nitrogen-rich structures whose properties depend on deposition conditions and elemental composition. Under suitable conditions, nitrogen can be incorporated into the face-centered-cubic (fcc) stainless-steel lattice, forming a metastable structure commonly referred to as expanded austenite (γN) or S-phase [16,17,18]. Nitrogen incorporation can produce lattice expansion and characteristic shifts in X-ray diffraction (XRD) peaks, particularly the (200) reflection. However, the crystallographic interpretation of these diffraction features remains complex because of lattice distortion, elastic anisotropy, stacking faults, and potentially related structural contributions [16,17,18,19,20,21,22,23,24]. The addition of titanium offers another means of modifying the composition and properties of stainless-steel nitride coatings because of its strong chemical affinity for nitrogen and its ability to form hard Ti–N bonds [25,26,27,28,29,30]. Previous research, including our 2019 study of Ti additions and deposition parameters in stainless-steel nitride coatings [31], has demonstrated that Ti incorporation can influence nitrogen uptake, microstructure, surface morphology, and hardness. Nevertheless, the specific influence of the relative stainless-steel/Ti sputtering contribution on the interconnected composition–structure–property relationships of SS–Ti–N coatings remains insufficiently clarified. When multiple deposition parameters are varied simultaneously, the effects of relative metallic flux, Ti incorporation, nitrogen enrichment, and microstructural evolution can be difficult to distinguish. To address this research gap, the present study systematically varies the stainless-steel target power while maintaining the Ti target power constant at 170 W. The stainless-steel power is reduced from 170 to 100 and 70 W, producing SS/Ti sputtering-power ratios of 1.00, 0.59, and 0.41, respectively. This design enables the influence of the relative target-power contribution to be examined without simultaneously changing the Ti target power. The novelty lies in systematically engineering the SS/Ti sputtering-power ratio and correlating it with Ti and N incorporation, lattice evolution, coating morphology, surface roughness, and mechanical hardness. Because these compositional and microstructural characteristics evolve together, the resulting property changes are evaluated as coupled composition–structure–property relationships rather than as independent effects of Ti or nitrogen. The coatings were characterized using X-ray photoelectron spectroscopy (XPS), XRD, scanning electron microscopy (SEM), stylus profilometry, and micro-Knoop hardness measurements. By correlating the sputtering-power ratio with composition, structural features, morphology, roughness, and hardness, this work provides insight into tailoring SS–Ti–N coatings for potential wear-resistant and protective surface-engineering applications.

## 2. Materials and Methods

### 2.1. Coating Deposition

**Figure 1:**
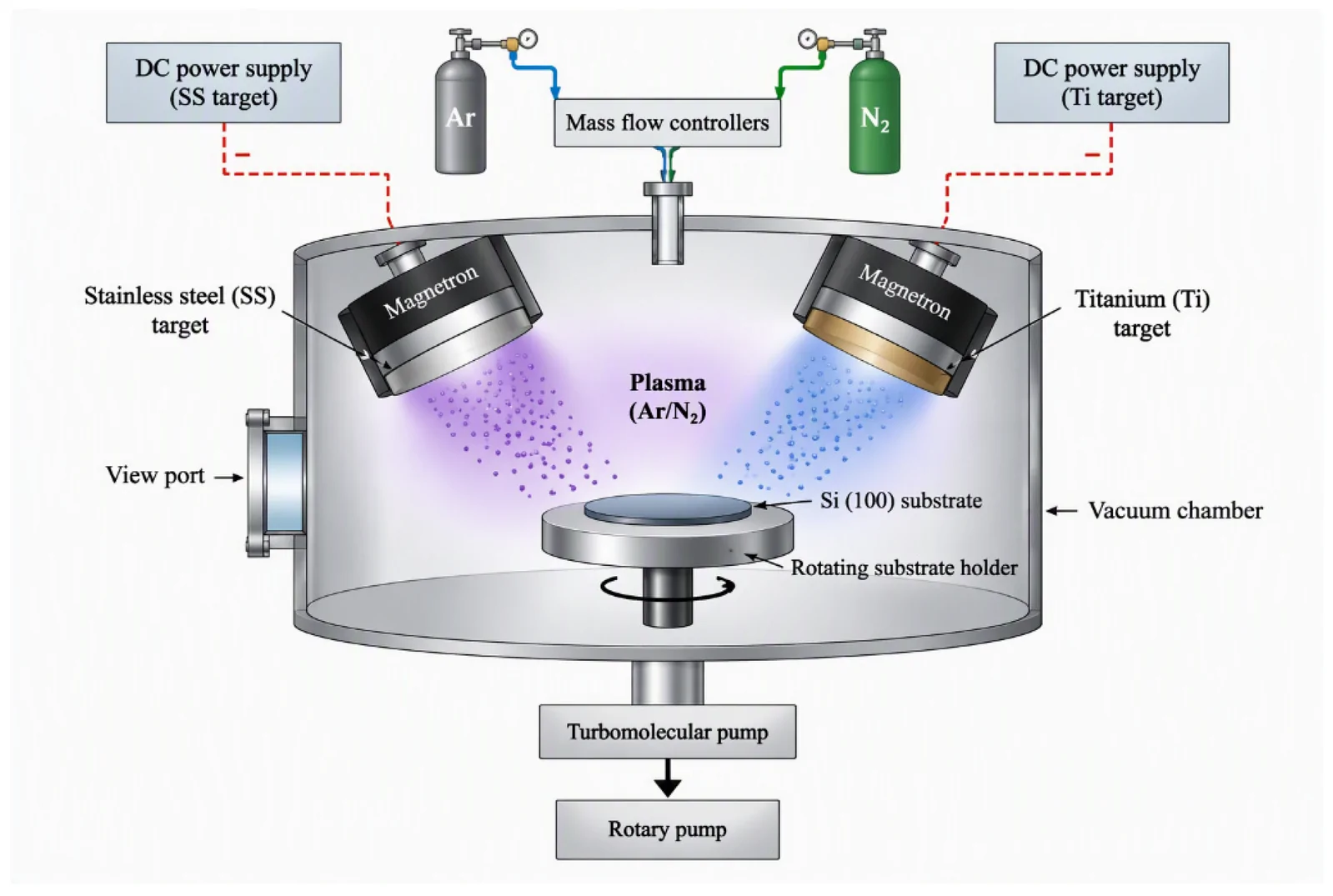
Schematic illustration of the reactive dual-target DC magnetron co-sputtering system used for deposition of SS–Ti–N coatings. Stainless-steel (SS) and titanium (Ti) targets were simultaneously sputtered in an Ar/N_2_ atmosphere onto Si (100) substrates mounted on a rotating substrate holder.

Commercial AISI 304 stainless-steel and high-purity titanium (99.99%) targets were used as sputtering sources to deposit SS–Ti–N coatings onto silicon wafers by reactive magnetron co-sputtering. The AISI 304 stainless-steel target had a nominal composition of 74 wt.% Fe, 18 wt.% Cr, and 8 wt.% Ni. Both targets were 50 mm in diameter and were operated using radio-frequency (RF) power supplies. Deposition was performed in a high-vacuum magnetron sputtering system with a base pressure of approximately (1 × 10^−6^ Torr (1.3 × 10^−4^ Pa). During sputtering of the functional coatings, the chamber working pressure was maintained at 5 mTorr (0.66 Pa). The substrate-to-target distance was approximately 9 cm, and the substrate temperature was maintained at 375 °C for all samples. Before deposition of the functional SS–Ti–N coatings, an approximately 50 nm metallic stainless-steel interlayer was deposited onto each silicon substrate to promote coating adhesion. The interlayer was deposited under an Ar atmosphere at a substrate bias of −50 V. Following interlayer deposition, the gas atmosphere was changed to Ar/N_2_ for reactive co-sputtering of the functional coating, using flow rates of 20 sccm Ar and 5 sccm N_2_. The total gas flow was maintained at 25 sccm, corresponding to an N_2_ fraction of 20%. Mass-flow controllers were used to regulate the gas flow rates. The substrate bias was set to −100 V for the subsequent deposition of the functional SS–Ti–N coatings, which was carried out for 3 h. Thus, the interlayer and functional coatings were deposited sequentially under different gas atmospheres and substrate biases. To systematically investigate the influence of the relative sputtering contributions of stainless steel and Ti, the Ti target power was maintained constant at 170 W, while the stainless-steel target power was varied among 170, 100, and 70 W. The corresponding stainless-steel/Ti sputtering-power ratios were 1.00, 0.59, and 0.41, respectively. The remaining specified coating-growth conditions were kept constant across the three samples to enable systematic comparison. The primary deposition parameters are summarized in Table 1 and a schematic diagram of the sputtering deposition system is shown in Figure 1. The coating thickness was monitored in situ using a quartz crystal microbalance and subsequently examined using cross-sectional scanning electron microscopy (SEM, Tescan Lyra FIB-SEM (TESCAN Group a.s., Brno, Czech Republic)). After 3 h of deposition, the functional coatings had a typical thickness of approximately 3 µm. Although the deposition approach shares methodological similarities with the earlier study by Alresheedi and Krzanowski [31], the present work focuses on a defined series of stainless-steel/Ti sputtering-power ratios while maintaining the Ti target power at 170 W. This experimental design provides a basis for systematically examining the relationships between the relative target-power contributions and the resulting coating composition, structure, morphology, surface roughness, and mechanical properties. The XPS-derived elemental compositions are presented separately in Table 2.

### 2.2. Characterization

The crystal structure of the deposited SS–Ti–N coatings was investigated by X-ray diffraction (XRD, Shimadzu Scientific Instruments, Inc., Columbia, MD, USA) using a Shimadzu XRD-6100 diffractometer equipped with Cu Kα radiation (*λ* = 1.54 Å). Diffraction patterns were recorded over the 2θ range of 30–65° with a step size of 0.05° and a scan speed of 10° min^−1^. The diffraction patterns were analyzed to identify the crystallographic structure and evaluate changes in lattice spacing associated with variations in coating composition. Lattice parameters were estimated from the principal fcc reflections using Bragg’s law and the standard relationship for a cubic crystal structure. The elemental composition of the coatings was determined by X-ray photoelectron spectroscopy (XPS) (XPS Axis HSi, Kratos Analytical, Manchester, UK). Measurements were performed using a monochromatic Mg Kα X-ray source operated at 15 kV and 10 mA. Survey spectra were acquired over a binding-energy range of 0–1200 eV using a pass energy of 160 eV, a step size of 1 eV, and a dwell time of 200 ms. Prior to spectral acquisition, the coating surfaces were sputter-etched using a 4 keV Ar^+^ ion beam to remove adventitious surface contamination. The Ti 2p, Fe 2p, Cr 2p, Ni 2p, N 1s, and O 1s core-level regions were analyzed to determine the elemental composition of the coatings. The concentrations were reported in atomic percent (at.%). The nitrogen-to-metal ratio, *N*/(*Fe* + *Cr* + *Ni* + *Ti*), was calculated from the measured atomic concentrations to evaluate the relative nitrogen incorporation into the coatings. The average compositional deviation was ±1.5 at.%, with a standard deviation of ±1.1 at.%; the corresponding uncertainty in the calculated N/Me ratio was determined from these compositional variations. The cross-sectional morphology and coating microstructure were examined using scanning electron microscopy (SEM). Cross-sectional specimens were prepared by mechanically fracturing the silicon substrates, exposing the coating cross sections for examination. SEM observations were performed using a Tescan Lyra FIB-SEM operated at an accelerating voltage of 6 keV. The cross-sectional images were used to evaluate the columnar growth morphology, grain structure, intercolumnar regions, and coating thickness. Surface roughness was measured using a Bruker Dektak stylus profilometer (Bruker Corporation (Nano Surfaces Division) in San Jose, CA, USA). The arithmetic average roughness (Ra) was used to quantify the surface topography of the coatings. Measurements were performed at five different locations on each coating surface, using a scan length of 4 mm and a cutoff length of 0.8 mm. The reported (Ra) value for each deposition condition was calculated by averaging the measurements obtained at these locations. The same measurement parameters were used for all samples to ensure consistency and enable reliable comparison of their surface roughness. The microhardness of the SS–Ti–N coatings was evaluated using a Wilson VH1202 microhardness tester equipped with a diamond Knoop indenter (Buehler (a division of Illinois Tool Works Inc./ITW) in Lake Bluff, IL, USA). Measurements were performed under a load of 10 gf with a dwell time of 15 s. Prior to indentation, the coating surfaces were prepared to ensure sufficient smoothness and cleanliness for reliable measurements. For each coating, ten individual indentations were made at well-separated locations to minimize the influence of local microstructural variations and interactions between adjacent indents. All measurements were performed using the same applied load and identical testing conditions for the three coatings. The Knoop hardness value for each indentation was determined from the measured indentation dimensions according to the instrument procedure. The ten individual measurements obtained for each coating were then used to calculate the arithmetic mean and standard deviation, and the results are reported as mean ± standard deviation. The use of multiple indentations provides a more representative measure of the coating hardness and allows the experimental scatter associated with local microstructural variations to be quantified.

## 3. Results and Discussion

### 3.1. Film Composition Analysis

To investigate the influence of the stainless-steel/Ti sputtering-power ratio on the chemical composition of the SS–Ti–N coatings, three deposition conditions were selected to progressively increase the relative contribution of Ti to the growing coatings. X-ray photoelectron spectroscopy (XPS, Kratos Analytical Ltd., Manchester, UK) was employed to determine the elemental composition and relative atomic concentrations of Fe, Cr, Ni, Ti, N, and O. The results are summarized in Table 2 and provide a quantitative basis for evaluating the compositional changes associated with systematically reducing the stainless-steel target power while maintaining the Ti target power at 170 W.

Sample 1, deposited using stainless-steel and Ti target powers of 170 and 170 W, respectively, exhibited the lowest Ti concentration among the three samples. The substrate bias was maintained at −100 V, and the deposition time was 3 h, resulting in a coating thickness of approximately 3 µm. XPS analysis revealed Fe, Cr, Ni, Ti, and N concentrations of 40.2, 10.6, 4.4, 4.25, and 35.6 at.%, respectively. Oxygen was also detected at 4.95 at.%. The Ti/(Fe + Cr + Ni) ratio was approximately 0.077, indicating a relatively low Ti contribution compared with the combined concentrations of Fe, Cr, and Ni. This result suggests that equal nominal sputtering powers did not produce equal relative concentrations of these metallic constituents in the coating. Differences in the sputtering characteristics and effective material fluxes from the two targets may contribute to this behavior. The nitrogen concentration of 35.6 at.% indicates substantial nitrogen incorporation under this deposition condition. The calculated N/metal ratio, defined as N/(Fe + Cr + Ni + Ti), was 0.60.
Sample 2, deposited using stainless-steel and Ti target powers of 100 and 170 W, respectively, exhibited an increased relative Ti contribution. The Ti concentration increased to 9.2 at.%, while the Fe, Cr, and Ni concentrations decreased to 30.8, 9.8, and 4.2 at.%, respectively. The corresponding Ti/(Fe + Cr + Ni) ratio increased to approximately 0.21. The nitrogen concentration also increased to 44.3 at.%, while the measured oxygen concentration decreased to 1.70 at.%. The calculated N/metal ratio increased to 0.82. These results demonstrate that reducing the stainless-steel target power while maintaining the Ti target power substantially modified the relative concentrations of the metallic constituents and nitrogen in the coating.
Sample 3, deposited using stainless-steel and Ti target powers of 70 and 170 W, respectively, exhibited the highest relative Ti contribution among the investigated conditions. The XPS-derived concentrations of Fe, Cr, Ni, Ti, and N were 24.5, 9.77, 4.03, 14.0, and 47.7 at.%, respectively. No detectable oxygen signal was observed under the measurement conditions. The Ti/(Fe + Cr + Ni) ratio increased to approximately 0.37, while the N/metal ratio reached 0.91. Thus, the lowest stainless-steel target power was associated with the highest measured Ti and N concentrations. The high nitrogen content is consistent with the nitrogen-rich, fcc-based structure observed by XRD. However, the composition should not be interpreted as that of a single stoichiometric TiN phase, because Fe, Cr, and Ni remain substantial constituents of the coating.

**Figure 2:**
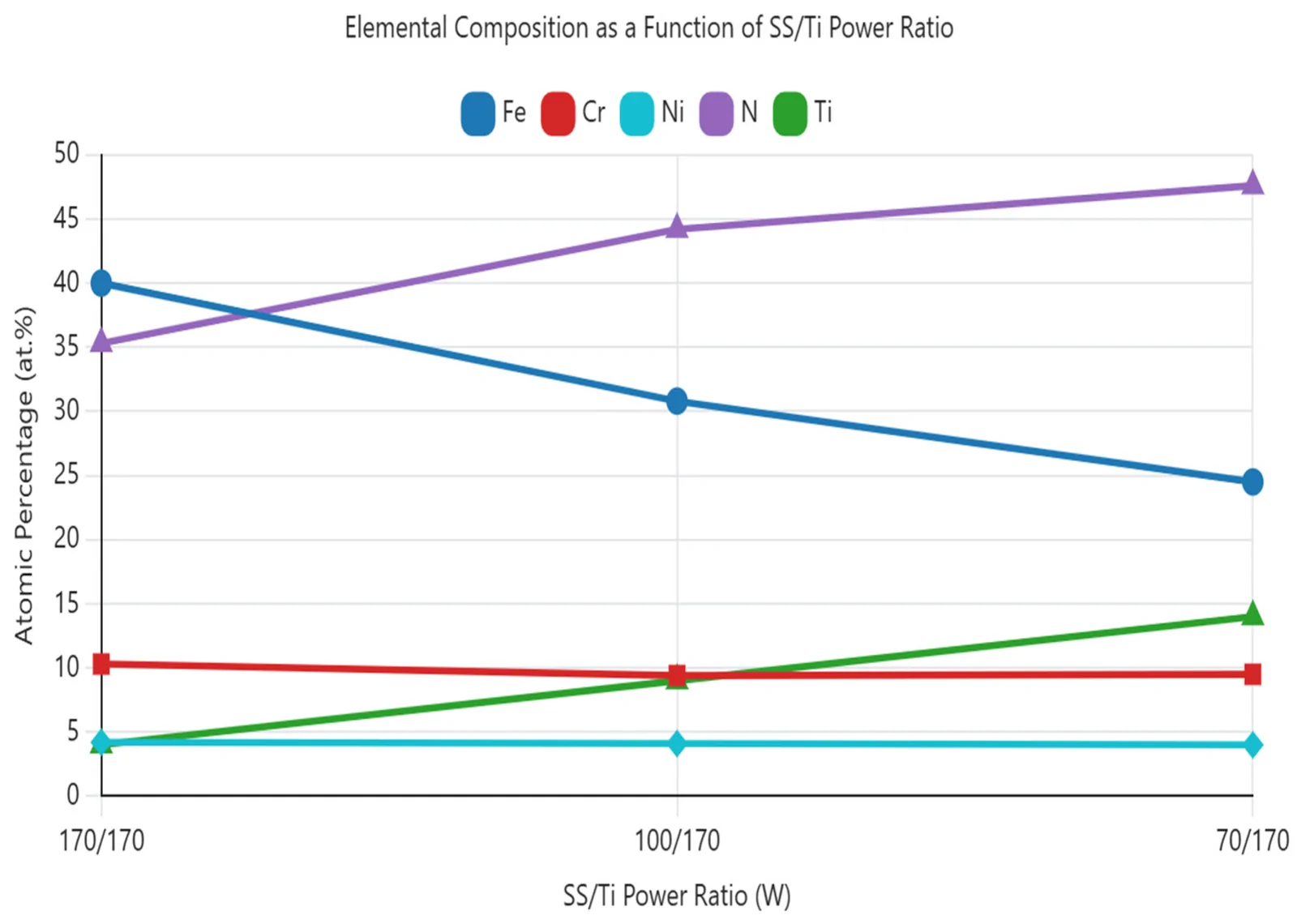
Atomic concentrations of Fe, Cr, Ni, Ti, and N in SS–Ti–N coatings as a function of the SS/Ti sputtering power ratio.

Reducing the stainless-steel target power from 170 to 100 and 70 W, while maintaining the Ti target power at 170 W, decreased the SS/Ti sputtering-power ratio from 1.00 to 0.59 and 0.41, respectively. Over this range, the Ti concentration increased from 4.25 to 9.2 and 14.0 at.%, while the nitrogen concentration increased from 35.6 to 44.3 and 47.7 at.%. As shown in Figure 2, these compositional trends were accompanied by an increase in the calculated N/metal ratio from 0.60 to 0.82 and 0.91. The results establish a clear association between the selected sputtering-power ratio and the relative incorporation of Ti and N. Nevertheless, because the metallic fluxes and nitrogen incorporation changed concurrently, their individual contributions to the observed compositional trends cannot be isolated from these measurements alone.

### 3.2. Crystal Structure

**Figure 3:**
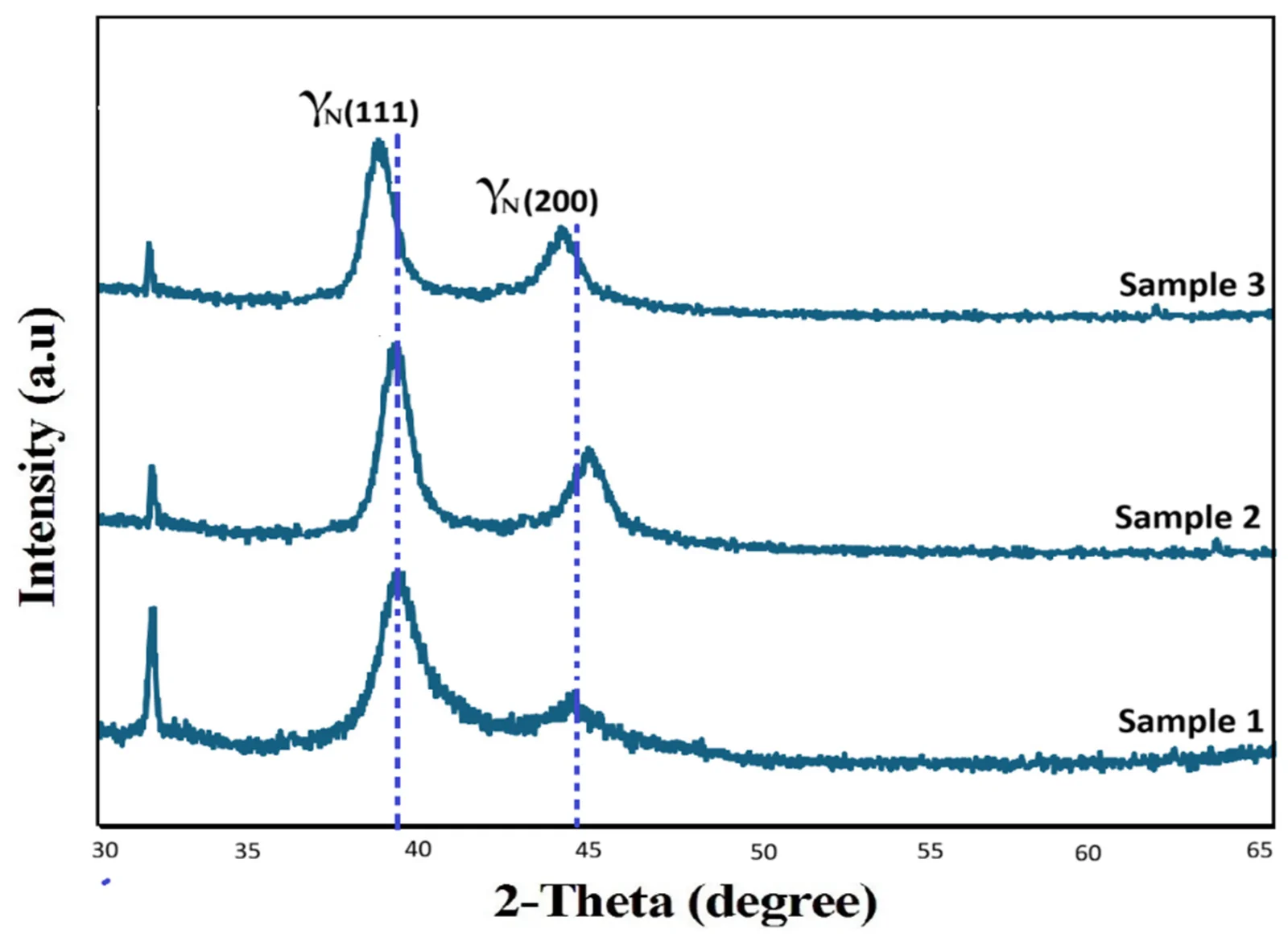
XRD patterns of the SS–Ti–N coatings deposited at different stainless-steel/Ti sputtering-power ratios: Sample 1 (170/170 W), Sample 2 (100/170 W), and Sample 3 (70/170 W). The diffraction peaks centered at approximately 39° and 45° were indexed to the nitrogen-expanded austenite phase (γN), corresponding to the γN (111) and γN (200) crystallographic planes, respectively.

The effect of the SS/Ti sputtering-power ratio on the crystal structure of the deposited SS–Ti–N coatings was investigated by X-ray diffraction (XRD). The diffraction patterns recorded over the (2θ) range of 30–65° are presented in Figure 3. 

All three coatings exhibit diffraction features that can be predominantly described on the basis of an fcc-type structure, with the principal observed reflections assigned to the (111) and (200) planes. The preservation of these fcc-type reflections indicates that the coatings maintain an fcc-based structural framework despite the systematic modification of the relative sputtering contributions from the stainless-steel and Ti targets. The observed diffraction behavior is consistent with a nitrogen-rich, fcc-based structure related to expanded austenite (γ_N_), commonly referred to as the S-phase) [32,33,34,35,36]. In nitrogen-containing austenitic stainless steels, interstitial incorporation of nitrogen can expand and distort the fcc metallic lattice, resulting in displacement of the diffraction peaks toward lower (2θ) values. Such peak shifts are commonly associated with nitrogen-induced lattice expansion in expanded-austenite systems. However, the diffraction behavior of nitrogen-containing stainless-steel coatings can be more complex than a simple uniform lattice expansion, particularly because residual stress, stacking faults, anisotropic lattice distortion, and peak-position anomalies can affect the observed reflections. This is particularly relevant to the (200) reflection in nitrogen-containing stainless-steel films. Therefore, the present XRD results are more appropriately described as an fcc-based nitrogen-rich structure related to expanded austenite, rather than as definitive evidence of a single stoichiometric nitride phase [34,35]. For comparison with the experimental diffraction patterns, a reference (γ_N_) pattern was calculated using the lattice parameter obtained from the experimentally observed (111) reflection. A systematic change in the diffraction pattern is observed as the relative Ti contribution increases. In particular, the (200) reflection progressively shifts toward lower (2θ) values as the deposition condition changes from 170 W SS/170 W Ti to 100 W SS/170 W Ti and finally to 70 W SS/170 W Ti. According to Bragg’s law,

nλ = 2dsinθ(1)

a displacement toward lower diffraction angles corresponds to an increase in the interplanar spacing (d). The observed shift therefore indicates a progressive modification and expansion of the lattice spacing as the SS/Ti sputtering-power ratio decreases.

This structural evolution is consistent with the compositional changes determined by XPS. As the SS target power decreases from 170 to 100 and 70 W while the Ti target remains at 170 W, the Ti concentration increases from 4.25 to 9.2 and 14.0 at.%, respectively, while the N concentration increases from 35.6 to 44.3 and 47.7 at.%. The increased nitrogen incorporation provides a plausible basis for the observed modification of lattice spacing because interstitial nitrogen can generate lattice strain and expansion. The increasing Ti incorporation may additionally modify the local chemical environment, bonding configuration, residual stress, and strain state of the growing coating [32,33,34,35,36]. Consequently, the observed peak evolution is considered a combined response to changes in nitrogen incorporation, Ti incorporation, and the relative metallic sputtering fluxes arriving at the substrate.

The lattice parameters were estimated from the experimentally measured positions of the (111) and (200) reflections using Bragg’s law and the fcc relationship:

(2) $$a_{\mathrm{hkl}}=d_{\mathrm{hkl}}\sqrt{h^{2}+k^{2}+l^{2}}$$

The calculated lattice parameters were approximately 0.406, 0.401, and 0.410 nm for the coatings deposited at SS/Ti target-power combinations of 170/170, 100/170, and 70/170 W, respectively. These values are larger than those typically reported for conventional austenitic stainless steel, indicating the formation of nitrogen-expanded austenite (γN). The enlarged lattice dimensions are consistent with the high nitrogen contents measured by XPS (35.6–47.7 at.%), although the lattice parameter does not exhibit a strictly monotonic dependence on nitrogen concentration, suggesting that additional factors such as residual stress, compositional variations, and lattice defects may also contribute to the observed lattice expansion [35]. It is important, however, that the lattice response cannot be attributed solely to nitrogen concentration. Reducing the stainless-steel target power while maintaining the Ti target at 170 W changes the relative fluxes of Fe, Cr, Ni, and Ti species reaching the substrate. The increasing Ti incorporation may alter the local bonding environment and strain state, while changes in deposition flux can also influence nucleation, growth kinetics, preferred orientation, and residual stress. The structural evolution observed in Figure 2 should therefore be regarded as the combined result of nitrogen incorporation, Ti incorporation, and changes in the relative metallic sputtering flux [37]. Differences between lattice parameters calculated from individual diffraction reflections may also arise from residual stress, lattice strain, preferred orientation [38,39,40,41,42], instrumental broadening, and peak overlap. Such effects are particularly relevant to sputtered coatings because non-equilibrium deposition conditions can introduce intrinsic stresses and structural defects. In expanded-austenite systems, reflection-dependent lattice parameters and anomalous shifts of the (200) reflection have also been reported, emphasizing the need for caution when interpreting individual peak positions.

### 3.3. Film Morphology

**Figure 4:**
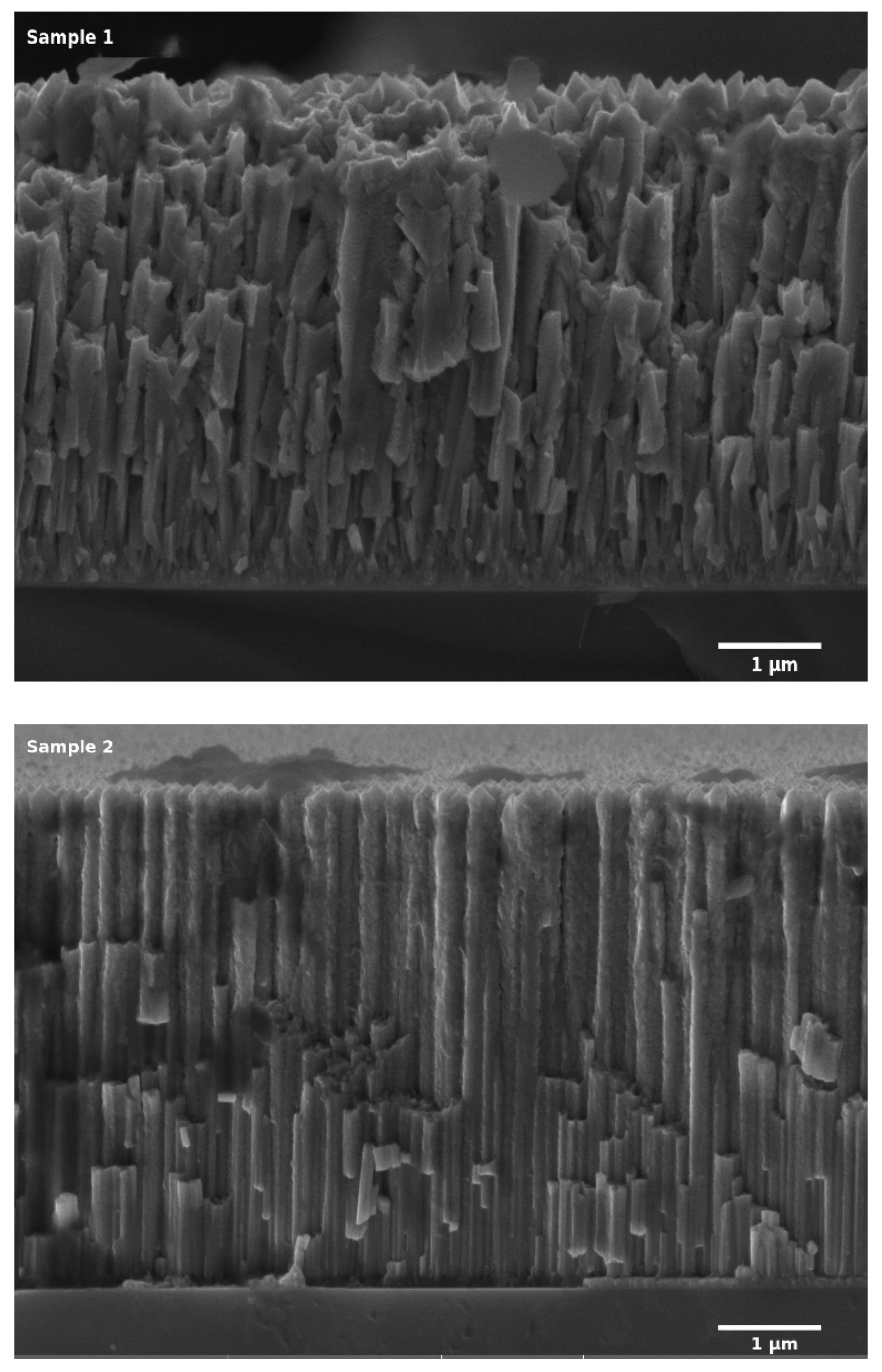

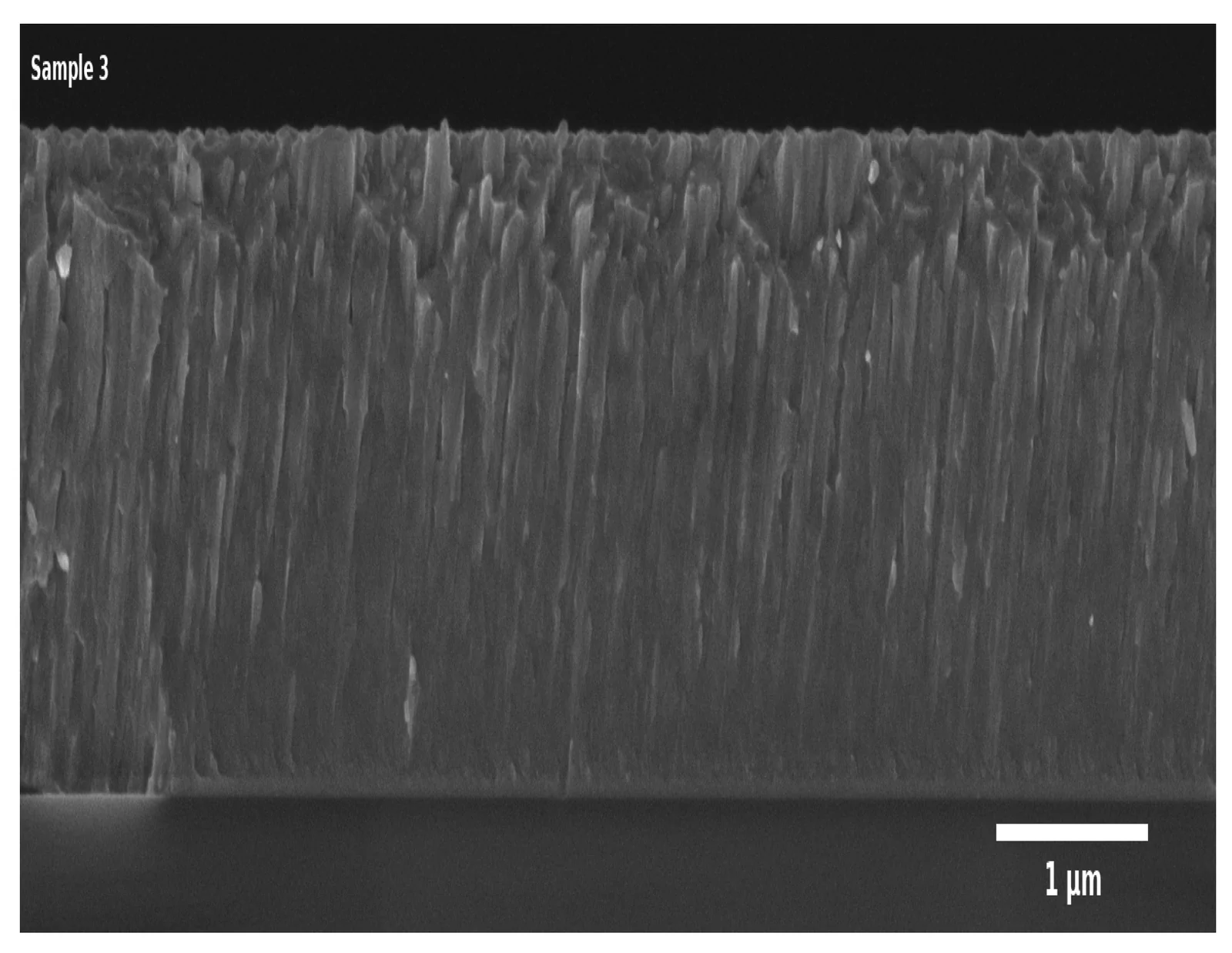
SEM cross-section of the SS–Ti–N coatings deposited at different stainless-steel/Ti sputtering-power ratios: Sample 1 (170/170 W), Sample 2 (100/170 W), and Sample 3 (70/170 W).

Cross-sectional scanning electron microscopy (SEM) images of the SS–Ti–N coatings deposited at different SS/Ti sputtering-power ratios are presented in Figure 4. The morphological evolution was examined by decreasing the stainless-steel (SS) target power from 170 to 100 and 70 W while maintaining the Ti target power at 170 W. This adjustment changes the relative contributions of the two targets to the deposited material and may consequently influence coating growth and columnar development. The coating deposited at 170 W SS/170 W Ti exhibits a predominantly columnar morphology characterized by relatively sharp, angular, and faceted features. Small wedge-shaped regions and intercolumnar voids are also visible, indicating a comparatively open columnar structure. Such features are consistent with competitive columnar growth commonly observed in sputtered coatings, although the specific growth mechanisms cannot be established from SEM images alone [43]. At 100 W SS/170 W Ti, the columns appear larger and more clearly developed than those in the 170/170 W coating. Although some intercolumnar voids remain, the columnar features appear more continuous and better defined. A further reduction in SS target power to 70 W produces a comparatively uniform and compact columnar morphology. In this coating, the sharp faceting and wedge-shaped features are less pronounced, and the columnar structure appears more regularly developed. Overall, the SEM observations indicate a progressive morphological change as the SS/Ti sputtering-power ratio decreases from 1.00 to 0.59 and 0.41. These morphological changes occur alongside systematic variations in the coating composition measured by XPS. As the SS/Ti sputtering-power ratio decreases, the Ti concentration increases from 4.25 to 9.2 and 14.0 at.%, while the N concentration increases from 35.6 to 44.3 and 47.7 at.%, respectively. The corresponding N/metal ratio, defined as (N/(Fe + Cr + Ni + Ti)), increases from 0.60 to 0.82 and 0.91. The coating deposited at 70 W SS/170 W Ti therefore exhibits both the highest measured Ti and N concentrations and the most uniform and compact columnar morphology among the investigated samples. The observed evolution may be associated with changes in the relative metallic fluxes and the incorporation of Ti and N, which can influence the structural development of multicomponent nitride coatings. However, because the composition and sputtering-power ratio change simultaneously, the individual contributions of Ti incorporation, nitrogen-related structural changes, and growth kinetics cannot be isolated from the present results [43,44]. The SEM images therefore establish a correlation between the deposition conditions, coating composition, and columnar morphology rather than demonstrating a single underlying mechanism.

### 3.4. Surface Roughness

**Figure 5:**
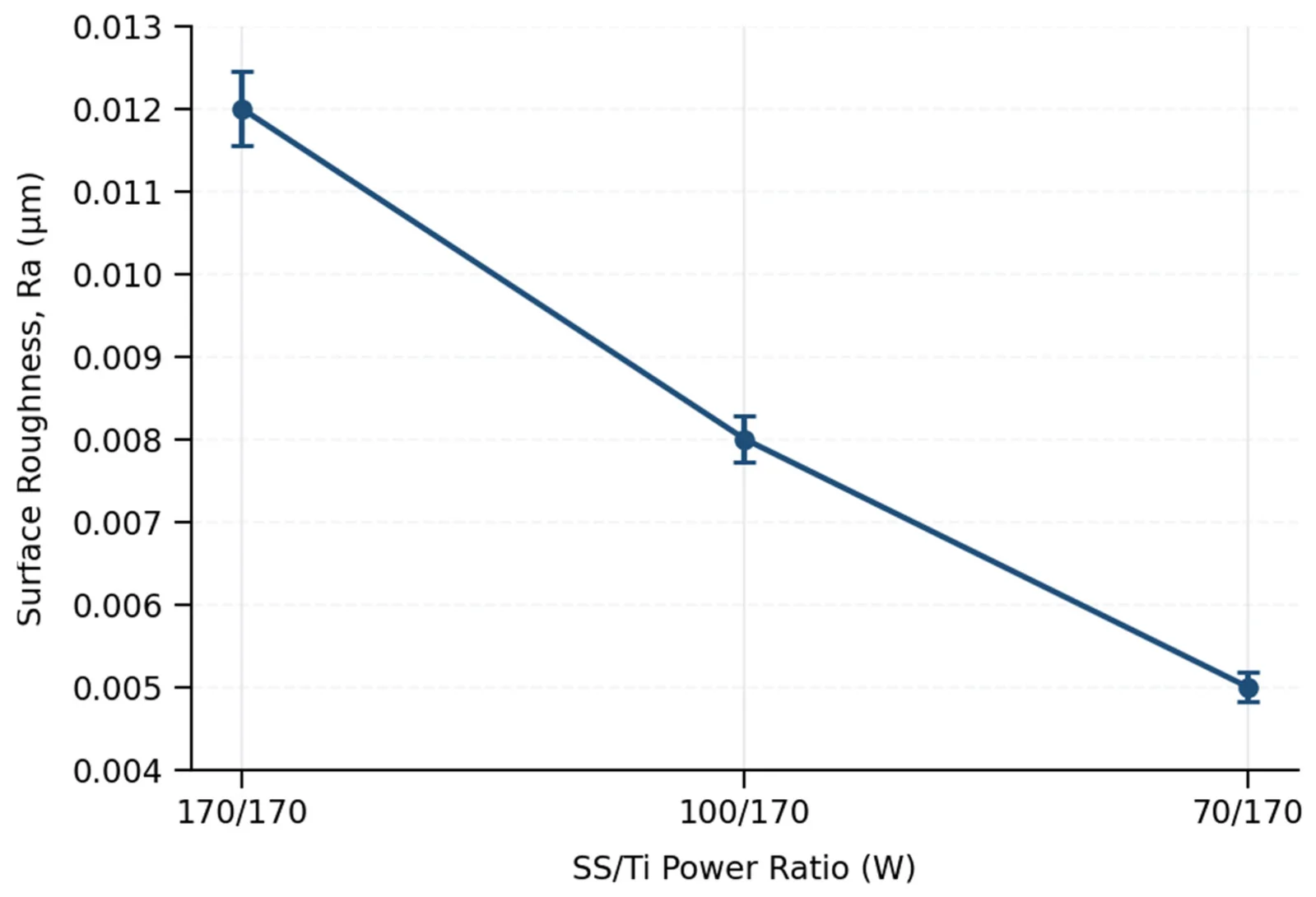
Surface roughness (*R_a_*) of the SS–Ti–N coatings as a function of the stainless-steel/Ti sputtering-power ratio. A systematic decrease in *R_a_* is observed from 0.0120 ± 0.00045 µm for the 170/170 W coating to 0.0080 ± 0.00028 µm for the 100/170 W coating and 0.0050 ± 0.00018 µm for the 70/170 W coating.

Figure 5 shows the arithmetic average surface roughness (Ra) of the SS–Ti–N coatings deposited at different stainless-steel/Ti sputtering-power ratios. Roughness was measured at five locations on each sample using a 4 mm scan length and a 0.8 mm cutoff length, with results reported as mean ± standard deviation. The (Ra) value decreases progressively from (0.0120 ± 0.00045) µm for the 170/170 W coating to (0.0080 ± 0.00028) µm for the 100/170 W coating and (0.0050 ± 0.00018) µm for the 70/170 W coating. This trend indicates that decreasing the stainless-steel target power while maintaining the Ti target power at 170 W is associated with a smoother coating surface [40,42]. The roughness trend is consistent with the cross-sectional SEM observations in Figure 3. The 170/170 W coating exhibits pronounced faceting and an open columnar structure with visible intercolumnar voids, whereas the 100/170 W coating displays more clearly developed columns. At 70/170 W, the morphology becomes comparatively uniform and compact, with less pronounced faceting and wedge-shaped features. The reduced prominence of these surface and columnar irregularities is consistent with the lower (Ra) value measured for this coating. These morphological differences may reflect changes in the relative fluxes of sputtered species and their influence on nucleation, adatom rearrangement, and competitive columnar growth. The increasing Ti and N concentrations observed by XPS as the stainless-steel target power decreases indicate that the chemical environment of the growing coatings also changes across the series. However, because the target-power ratio, elemental composition, and microstructure vary concurrently, the individual contributions of Ti incorporation, nitrogen incorporation, and growth-related processes cannot be isolated from the present measurements. The decrease in surface roughness is therefore best interpreted as part of a coupled composition–microstructure response to the variation in the stainless-steel/Ti sputtering-power ratio, rather than as the effect of a single compositional factor.

### 3.5. Hardness

**Figure 6:**
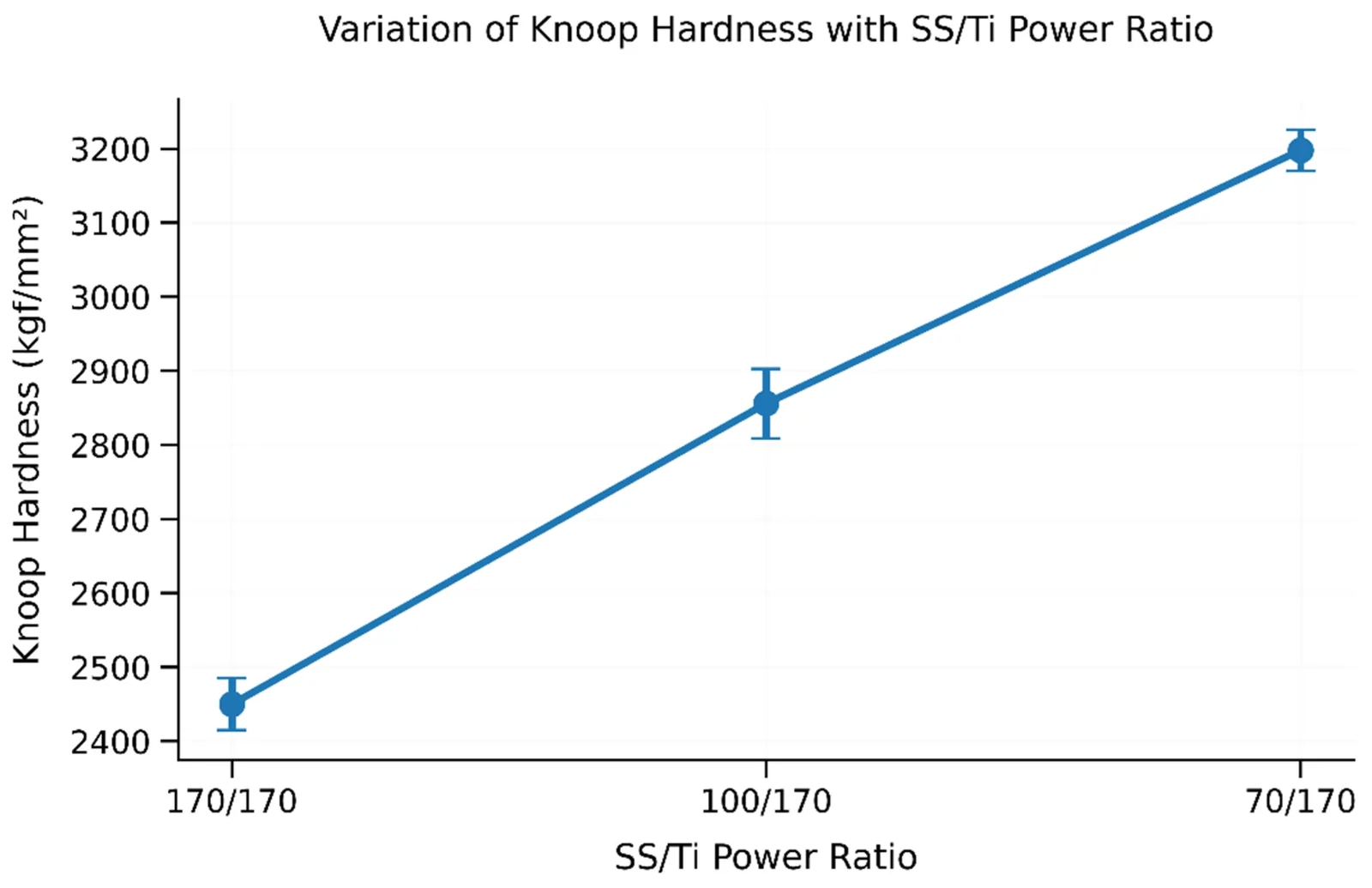
Variation in Knoop hardness of the SS–Ti–N coatings as a function of the SS/Ti sputtering-power ratio. Data points represent the mean hardness obtained from ten indentations, and error bars indicate ± standard deviation.

The Knoop hardness of the SS–Ti–N coatings was evaluated using a microhardness tester. Figure 6 shows the variation in hardness with the stainless-steel/Ti sputtering-power ratio. As the stainless-steel target power decreases while the Ti target power remains constant at 170 W, the average hardness increases from (2450 ± 35) kgf/mm^2^ for the 170/170 W coating to (2856 ± 47) kgf/mm^2^ for the 100/170 W coating and (3200 ± 28) kgf/mm^2^ for the 70/170 W coating. The maximum value corresponds to approximately 31.4 GPa, indicating a substantial change in mechanical response across the investigated deposition conditions. The achieved hardness (3200 HK) was also reported in the author’s recently granted Saudi patent (SA 1020251601) [45]. The hardness increase is accompanied by changes in coating composition and structure. XPS measurements show that the Ti concentration increases from 4.25 to 14.0 at.% and the N concentration from 35.6 to 47.7 at.% as the stainless-steel target power decreases from 170 to 70 W. Over the same range, the N/metal ratio, defined as (N/(Fe + Cr + Ni + Ti)), increases from 0.60 to 0.91. These compositional changes occur alongside variations in the XRD patterns, columnar morphology, and surface roughness. Because the relative sputtering contributions change the incorporation of multiple elements simultaneously, the hardness trend cannot be attributed exclusively to Ti or nitrogen. Several mechanisms may contribute to the observed strengthening. Increased nitrogen incorporation may produce lattice distortion in the nitrogen-rich fcc-based structure, potentially increasing resistance to plastic deformation. The increasing Ti concentration may also modify the local bonding environment and structural characteristics of the multicomponent coating. In addition, cross-sectional SEM reveals a morphological transition from a relatively open, faceted columnar structure in the 170/170 W coating to a comparatively uniform and compact columnar structure in the 70/170 W coating. This morphological evolution may contribute to the measured hardness by providing a more continuous structure that resists localized deformation. The accompanying decrease in surface roughness from (0.0120 ± 0.00045) to (0.0050 ± 0.00018) µm is consistent with the observed change in surface and cross-sectional morphology. Similar relationships between composition, microstructure, and hardness have been discussed for sputtered stainless-steel–Ti–N coatings [46,47]. These interpretations should nevertheless be considered in the context of the experimental design. Although the three coatings exhibit a clear correlation between the SS/Ti sputtering-power ratio and hardness, the individual contributions of Ti incorporation, nitrogen-related structural changes, and morphological evolution cannot be quantitatively separated. Furthermore, although the coating thickness was approximately 3 µm for all samples, thickness-related substrate effects during indentation cannot be entirely excluded. The hardness enhancement is therefore best interpreted as a coupled composition–microstructure response to the controlled variation of the SS/Ti sputtering-power ratio rather than as evidence for a single dominant strengthening mechanism.

## 4. Conclusions

SS–Ti–N coatings were deposited by reactive magnetron sputtering to investigate how the stainless-steel/Ti sputtering-power ratio influences their composition, morphology, surface roughness, and mechanical hardness. With the Ti target power maintained at 170 W, decreasing the stainless-steel target power from 170 to 100 and 70 W was accompanied by increased Ti and nitrogen incorporation, changes in columnar morphology, reduced surface roughness, and increased Knoop hardness. Among the investigated coatings, the 70/170 W condition exhibited the lowest arithmetic average roughness (Ra = 0.0050 ± 0.00018) µm) and the highest Knoop hardness (approximately 31.4 GPa). The combined results demonstrate that adjusting the relative sputtering-power contributions of stainless steel and Ti provides a practical approach for tailoring the measured surface and mechanical properties of SS–Ti–N coatings. The simultaneous changes in elemental composition and morphology indicate that the observed property improvements reflect a coupled composition–microstructure response; the individual contributions of Ti, nitrogen, and microstructural evolution cannot be independently quantified from the present experiments. Although the high hardness and low surface roughness of the optimized coating are promising for protective surface-engineering applications, direct wear and scratch tests are still required to its tribological performance and coating adhesion. These findings provide a basis for further optimization of multicomponent nitride coatings through controlled adjustment of metallic target contributions.

## Figures and Tables

**Table 1:** Main deposition parameters.

Deposition Parameter	Sample 1	Sample 2	Sample 3
Stainless-steel target power (W)	170	100	70
Ti target power (W)	170	170	170
SS/Ti sputtering-power ratio	1.00	0.59	0.41
Base pressure (Torr)	1 × 10^−6^	1 × 10^−6^	1 × 10^−6^
Working pressure during deposition (mTorr)	5	5	5
Ar flow rate (sccm)	20	20	20
N_2_ flow rate (sccm)	5	5	5
Substrate temperature (°C)	375	375	375
Deposition time (h)	3	3	3
Thickness (µm)	~3	~3	~3

**Table 2:** XPS-derived elemental composition of the SS-Ti-N coatings deposited at different SS/Ti sputtering power ratios.

SS/Ti Power (W)	At.% Fe	At. % Cr	At.% Ni	At.% N	At.% O_2_	At.% Ti	N/ (Fe + Cr + Ni + Ti)
170/170	40.2	10.6	4.4	35.6	4.95	4.25	0.60
100/170	30.8	9.8	4.2	44.3	1.7	9.2	0.82
70/170	24.5	9.77	4.03	47.7	ND	14	0.91

## Data Availability

The original contributions presented in this study are included in the article. Further inquiries can be directed to the corresponding author.
